# Comment on Alayat et al. The Effectiveness of Photobiomodulation Therapy on Pain and Function in Patients with Patellofemoral Pain Syndrome—A Systematic Review and Meta-Analysis. *J. Clin. Med.* 2026, *15*, 20

**DOI:** 10.3390/jcm15176635

**Published:** 2026-08-28

**Authors:** Hernán Andrés de la Barra Ortiz, Claudio Hernán Chamorro Lange

**Affiliations:** Exercise and Rehabilitation Sciences Institute, School of Physical Therapy, Faculty of Rehabilitation Sciences, Universidad Andres Bello, Santiago 7591538, Chile; claudio.chamorro@unab.cl

We read with interest the systematic review and meta-analysis by Alayat et al. [1], published in the Journal of Clinical Medicine, examining the effects of photobiomodulation therapy (PBM) on pain and function in patellofemoral pain syndrome (PFPS), a condition that accounts for up to 25% of sports medicine consultations and approximately 20–40% of cases managed in specialized clinics [2,3]. We appreciate the authors’ effort to bring together the available evidence. However, several methodological and statistical issues warrant closer consideration, as they may affect the interpretation and clinical relevance of the findings.

First, in trials with multiple intervention arms, some studies appear to contribute more than one comparison to the same meta-analysis. This issue can be observed, for example, in the pain forest plot for Özlü et al. [4] and in the functional outcome forest plot for Özlü et al. [4] and Nouri et al. [5]. When multiple intervention arms from a single trial are included, the Cochrane Handbook for Systematic Reviews of Interventions recommends specific strategies to avoid unit-of-analysis errors and correlated comparisons, such as combining intervention groups, selecting a single pairwise comparison, splitting the shared control group, explicitly accounting for correlations, or conducting a network meta-analysis [6,7,8]. Given the stated adherence to Cochrane guidance, clarification on this point would strengthen confidence in the pooled estimates.

Second, although grouping studies as “PBM plus exercise versus corresponding controls” is reasonable, the clinical content of these groups varies considerably [1]. In some trials, PBM was delivered alongside exercise and manual therapy, such as patellar mobilization [9]. In others, it formed part of broader multimodal physiotherapy programs that included soft-tissue techniques, electrotherapy, or taping [10]. These interventions are not equivalent and may have independent effects on pain and function, making it difficult to isolate the specific contribution of PBM. Several studies also compared active PBM plus exercise with sham PBM plus exercise within multimodal approaches, including Gavish et al. [10], Goyal [11], Qayyum et al. [9], and Özlü et al. [4], or with no treatment, as in Pocai et al. [12], which could have allowed more clinically focused subgroup analyses.

Third, according to Table 1, pain was reported as being assessed using the visual analog scale (VAS) across all included trials, including Nouri et al. [5], Pocai et al. [12], Gavish et al. [10], Goyal [11], Qayyum et al. [9], Eurcherdkul et al. [13], Özlü et al. [4], and Allam et al. [14]. In this context, pooling results as mean differences (MD), rather than standardized mean differences (SMD), would likely be more transparent and clinically interpretable, particularly when the same instrument is used across studies to assess the same outcome, as in the case of the VAS [15]. While SMDs are useful when outcomes are measured on different scales, their use when a common scale is available may reduce interpretability, introduce additional heterogeneity, and has been shown to yield poorer statistical performance and greater uncertainty compared with MDs in some scenarios [15,16]. When effects are expressed as SMDs, this comparison becomes less intuitive unless results are back-transformed [17]. This point is particularly relevant because the authors indicate that imprecision was judged against minimally important differences defined on the original scale (1.5–2.0 points) [18]. A similar issue applies to functional outcomes, as most studies used the Kujala Anterior Knee Pain Scale (AKPS) [18]. Moreover, the confidence intervals for both pain (SMD −0.83; 95% CI −1.40 to −0.27) and function (SMD 0.68; 95% CI 0.08 to 1.27) are relatively wide, suggesting substantial uncertainty in the true magnitude of the effects, particularly in the context of meta-analyses with limited numbers of trials and events, where wide confidence intervals reflect instability in the pooled estimates [19]. Notably, although the authors acknowledge in the text that pain was assessed using either the VAS in Nouri et al. [5], Pocai et al. [12], Gavish et al. [10], Qayyum et al. [9], Eurcherdkul et al. [13], Özlü et al. [4], and Allam et al. [14], or the numeric rating scale (NRS) in Goyal [11], which may have served as a justification for the use of the SMD, Table 1 reports the VAS as the pain assessment instrument across all included studies, without distinguishing that one study assessed pain using the NRS [1]. This internal inconsistency may have reinforced an implicit assumption of scale equivalence and contributed to the choice of SMD-based pooling. From a clinical perspective, although VAS and NRS are both intended to quantify pain intensity and often show moderate to high correlations, correlation does not imply measurement equivalence or agreement [20,21]. Agreement-based analyses have demonstrated systematic bias and wide limits of agreement between these instruments, indicating that they are not directly interchangeable [20,21,22]. Moreover, VAS is typically treated as a continuous measure, whereas NRS is a discrete ordinal scale [20,22], and both instruments present distinct minimally clinically important differences (MCIDs), with reported values of approximately 15–20 mm for VAS and 1.3–2.0 points for NRS, depending on the population and clinical context [23,24]. Consequently, combining VAS and NRS under an assumption of equivalence remains methodologically suboptimal and may compromise the clinical interpretability and validity of pooled effect estimates.

Fourth, functional outcomes derived from different instruments, such as the Kujala Anterior Knee Pain Scale (AKPS) in Nouri et al. [5], Pocai et al. [12], Gavish et al. [10], Eurcherdkul et al. [13], Özlü et al. [4], and Allam et al. [14], and the Western Ontario and McMaster Universities Osteoarthritis Index (WOMAC) in Nouri et al. [5] and Goyal [11], appear to have been pooled in the functional forest plot. These scales capture different constructs—AKPS assesses function related to anterior knee pain, where higher scores indicate better function, and WOMAC assesses disability, where improvements are reflected by lower scores [25,26]—and their combination may increase heterogeneity and indirectness. In addition, the direction of the AKPS appears inconsistent in at least one study, as observed in Eurcherdkul et al. [13], where negative values are reported despite higher scores conventionally indicating better function. This variation raises the possibility of issues related to score coding or transformation.

Fifth, the review includes heterogeneous patient populations, PBM modalities (low- and high-intensity laser therapy), dosimetry parameters, application protocols, and participant characteristics. Exploring more homogeneous subgroups based on intervention type (e.g., PBM plus exercise versus placebo plus exercise versus standard physiotherapy) or separating PBM modalities could help clarify which patients and treatment protocols are most likely to benefit.

Finally, given the rapid expansion of the PBM literature, extending the search beyond January 2025, as stated in the abstract, could help ensure that recently published trials are not missed.

In summary, clearer reporting on the handling of multi-arm trials, the heterogeneity of co-interventions, the choice of effect measures, and the pooling of functional outcomes would strengthen the methodological rigor and improve the clinical interpretation of this otherwise valuable review.

## Data Availability

No new data were created or analyzed in this study. Data sharing is not applicable to this article.

## Abbreviations

The following abbreviations are used in this manuscript:

AKPS	Anterior Knee Pain Scale (Kujala Score)
MD	Mean Difference
MCID	Minimally Clinically Important Difference
PBM	Photobiomodulation (therapy)
PFPS	Patellofemoral Pain Syndrome
RCT	Randomized Controlled Trial
SMD	Standardized Mean Difference
VAS	Visual Analog Scale
WOMAC	Western Ontario and McMaster Universities Osteoarthritis Index
